# Serpin-loaded extracellular vesicles promote tissue repair in a mouse model of impaired wound healing

**DOI:** 10.1186/s12951-022-01656-7

**Published:** 2022-11-05

**Authors:** Dong Jun Park, Erika Duggan, Kayla Ho, Robert A. Dorschner, Marek Dobke, John P. Nolan, Brian P. Eliceiri

**Affiliations:** 1 Departments of Surgery, University of California San Diego, 9500 Gilman Drive, MC 8236, La Jolla, CA 92093-8236 USA; 2 Dermatology, University of California San Diego, 9500 Gilman Drive, MC 8236, La Jolla, CA 92093-8236 USA; 3 Scintillon Institute, 6868 Nancy Ridge, San Diego, CA 92121 USA

**Keywords:** Extracellular vesicles, Exosomes, Wound healing, Diabetes, Serpin, Extracellular matrix

## Abstract

Chronic metabolic diseases such as diabetes are characterized by delayed wound healing and a dysregulation of the inflammatory phase of wound repair. Our study focuses on changes in the payload of extracellular vesicles (EVs) communicating between immune cells and stromal cells in the wound bed, which regulate the rate of wound closure. Adoptive transfer of EVs from genetically defined mouse models are used here to demonstrate a functional and molecular basis for differences in the pro-reparative biological activity of diabetic (db/db) vs. wildtype EVs in wound healing. We identify several members of the Serpin family of serine protease inhibitors that are absent in db/db EVs, then we overexpress Serpin A1, F2 and G1 in EVs to evaluate their effect on wound healing in db/db mice. Serpins have an important role in regulating levels of elastase, plasmin and complement factors that coordinate immune cell signaling in full thickness wounds in a diabetic model. Here, we establish a novel therapeutic approach by engineering the payload of EVs based on proteomic analysis. Serpin-loaded EVs were used to rescue the Serpin deficiency identified by proteomics and promote wound healing in db/db mice, as well as evaluated how EVs affected extracellular matrix remodeling and the resolution of tissue injury. Therefore, we propose that the identification of EV payloads that are downregulated in diabetic wounds can be systematically analyzed for their functional activity and potential as a therapeutic, based on whether their re-expression in engineered EVs restores normal kinetics of tissue repair in chronic wounds.

**Graphical Abstract:**

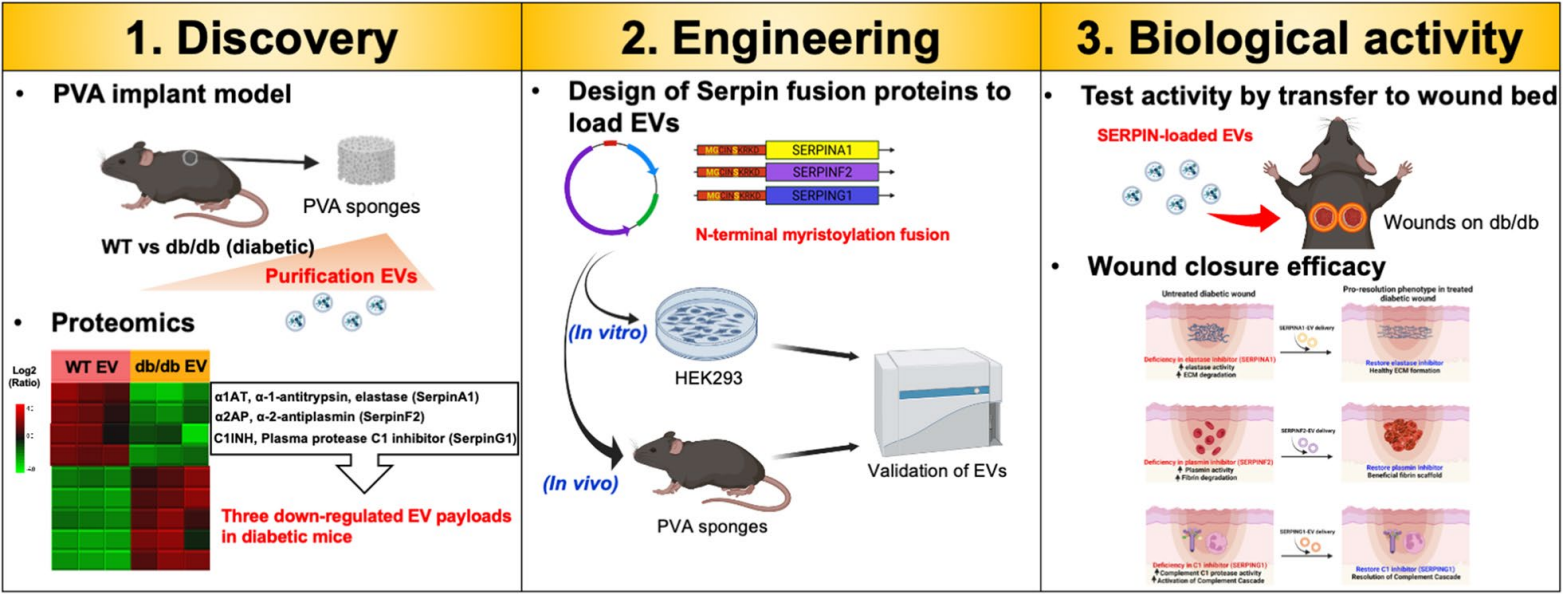

**Supplementary Information:**

The online version contains supplementary material available at 10.1186/s12951-022-01656-7.

## Background

Healthy wound repair is characterized by direct and indirect intercellular interactions between cell types that mediate hemostasis, inflammation, proliferation and remodeling [1, 2]. Increasing evidence suggests that extracellular vesicles (EVs) mediate indirect signaling between cell types that is pro-reparative in wound healing [3–5]. Studies of pathways regulating EV biogenesis have demonstrated that the cellular source of EVs affects their formation, payload, and biological activity in the wound bed [6]. For example, platelet-derived EVs promote coagulation in hemostasis, while neutrophil-derived EVs regulate the expression of adhesion factors on the endothelium [7, 8]. As a wound transitions from the inflammatory phase to proliferation and re-epithelialization, macrophage-derived EVs drive macrophage polarization to an anti-inflammatory phenotype to mediate signaling between wound-edge fibroblasts and keratinocytes to promote wound closure [9]. The resolution of the inflammatory phase of wound healing involves re-epithelialization through keratinocyte migration and remodeling of granulation tissue into more permanent extracellular matrix (ECM) through expression of collagen and proteoglycans and regulation of the secretion of proteases [10–12]. In vitro studies indicate that the remodeling phase of wound healing can be stimulated by EV-mediated signaling such as in the use of mesenchymal stem cell-derived EVs that accelerate closure of chronic wounds by establishing a pro-reparative microenvironment [13, 14].

Diabetes is a chronic metabolic disease that is complicated by delayed wound healing and dysregulation of the inflammatory phase wound repair [15–19], leading to chronic wounds and substantial morbidity. To identify secreted factors in the diabetic wound bed associated with delayed cutaneous wound closure as potential therapeutic targets, we used mice lacking the Leptin receptor (Lepr^−/−^; db/db), which is a Type II diabetic model that is hyperglycemic and presents with a well-defined phenotype of delayed cutaneous wound closure [20–23]. We used sterile subcutaneous PVA sponge implants in mice as a source for EVs based on a recruitment of neutrophils, monocytes and macrophages similar to the inflammation phase of the wound healing [24–26]. This approach facilitated efficient EV collection that avoided cell culture artifacts including EVs that are unrelated to the wound bed, as well as artifacts associated with tissue disruption, and was compatible with species-matched genetic studies of EV biological activity. We applied this approach to genetically-defined models of impaired wound healing and report a unique a signature of EVs from the wound bed of db/db mice based on their protein payload and relevance to the inflammation phase of wound healing [25, 27].

Here we have identified several members of the Serpin family of serine protease inhibitors that were down-regulated in db/db vs. wildtype (WT) EVs, which we then re-expressed, promoted wound closure. Serpins are of particular interest in wound healing because of their inhibitory effects on specific proteases that are relevant in inflammation and ECM remodeling that affects cell migration and proliferation that is a hallmark of the wound response.

Members of the Serpin superfamily regulate blood pressure, hormone transport, insulin sensitivity and the inflammatory response [28]. The identification of Serpin deficiencies in diabetic wounds suggests the therapeutic potential of re-expression of these Serpins to promote tissue repair by modulating protease activity and inflammation responses. In combination with the emerging importance of EVs as therapeutic nanocarriers for proteins and nucleic acids in complex animal systems, our findings suggested that Serpin-loaded EVs can have a therapeutic potential in promoting closure of chronic wounds [29].

## Results

### Subcutaneous sponge implants as an in vivo source of EVs for allotransplantation

**Fig. 1:**
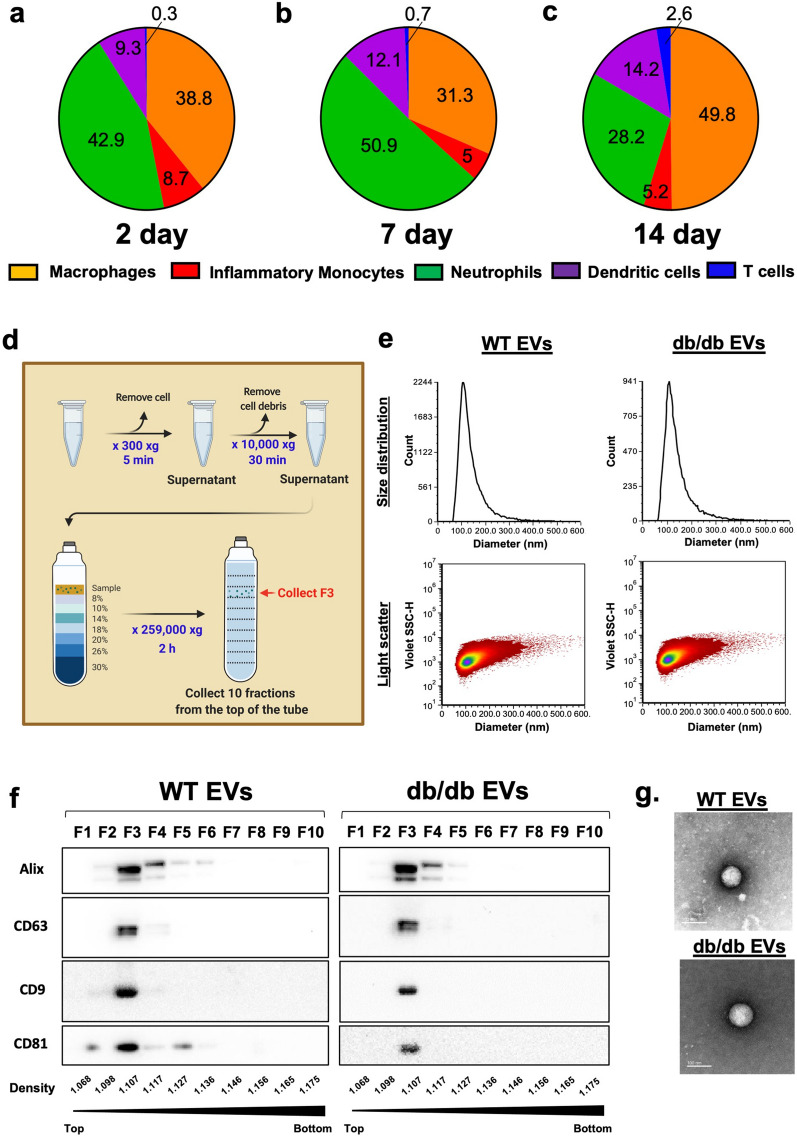
Profile of cell recruitment and EV analysis in the implant model. **a** Flow cytometry analysis of cell types recruited to PVA sponges 2 days post-implantation focusing on the macrophages (orange), inflammatory monocyte (red), neutrophils; (green), dendritic cells (purple), and T cells (blue). Mean + SD, n = 7 independent experiments with supporting statistical analysis in Fig S1. **b** Mean cell type distributions in the PVA sponges 7 days post-implantation. Mean + SD, n = 4 independent experiments, t-test comparison test. **c** Mean cell type distribution of cell types in PVA sponges 14 days post-implantation. Mean + SD, n = 10 independent experiments, t-test comparison test (Supporting statistical analysis shown in Supplementary Fig. 1). **d** Schematic of EV isolation workflow based on density gradient ultracentrifugation. **e** A representative example of the validation studies defining size distribution and light scatter on WT and db/db EVs by vFRed analysis. **f** Immunoblot showing the specific EV markers (CD81, CD9, CD63, and Alix) in the density gradient fractions on the WT vs. db/db EVs. n = 6 independent experiments. **g** Transmission electron microscopy of WT vs. db/db EVs at 100 k magnification, size bar = 100 nm. All representative images showed as observed in three independent experiments

Implants of sterile polyvinyl alcohol (PVA) sponge elicited the recruitment of neutrophils and macrophages relevant in the inflammatory phase of wound healing, therefore, wound fluid from these implants were analyzed for cellular and EV content (Fig. 1). Immunophenotyping by flow cytometry of wound fluid cells collected over a time course of 2–14 days identified an infiltration of leukocytes such as macrophages (CD45^+^, CD11b^+^, F4/80^+^), inflammatory monocytes (CD45^+^, Ly6c^+^, CD11b^+^), dendritic cells (DCs, CD45^+^, CD11c^+^, MHCII^+^, CD11b^+^), neutrophils (CD45^+^, Ly6G^high^, Ly6c^int^), and T cells (CD4^+^). Neutrophils and macrophages predominated at each time point (Fig. 1a–c) followed by DCs, inflammatory monocytes and T cells. In combination with Supplementary Data showing statistical analysis (Additional file 1: Fig. S1), expression of canonical EV biogenesis genes (Additional file 1: Fig. S2 and Table. S1) and cell profiling of WT vs. db/db mice (Additional file 1: Fig. S3), these studies defined the immune cell phenotype of the implant model used as an in vivo EV source relevant to the inflammatory phase of wound healing. To characterize the EV released in the PVA sponge model, EVs were enriched by density ultracentrifugation (Fig. 1d) and analyzed by vesicle flow cytometry to demonstrate a similar EV size distribution (Fig. 1e), concentration, expression of canonical EV markers (Fig. 1f), and appearance by transmission electron microscopy (Fig. 1g) in WT and db/db EVs that was supported by vesicle flow cytometry (vFC) analysis based on MISEV2018 standards (Additional file 1: Fig. S4). Quantitative analysis of WT and db/db EVs using vFC demonstrates consistency in EV size (Additional file 1: Fig. S4 a and g) and light scatter (Additional file 1: Fig. S4 b and h). Similarly, EVs from WT and db/db mice were comparable for their staining for EV membrane phosphatidylserine using Annexin V (Fig. S4 c and i) and expression of the tetraspanins CD9, CD63 and CD81 (TS) on the EV surface (Fig. S4 d and j) with normalized expression of Annexin-V and TS expressed as histograms (Additional file 1: Fig. S4 e,f,k,l). Together, these representative plots demonstrate the analytical power of vFC for quantitative analysis of complex EV mixtures from biological fluids such as the PVA sponge and wound fluid.

### Pro-reparative capacity of EVs defined by genetics of donor

**Fig. 2:**
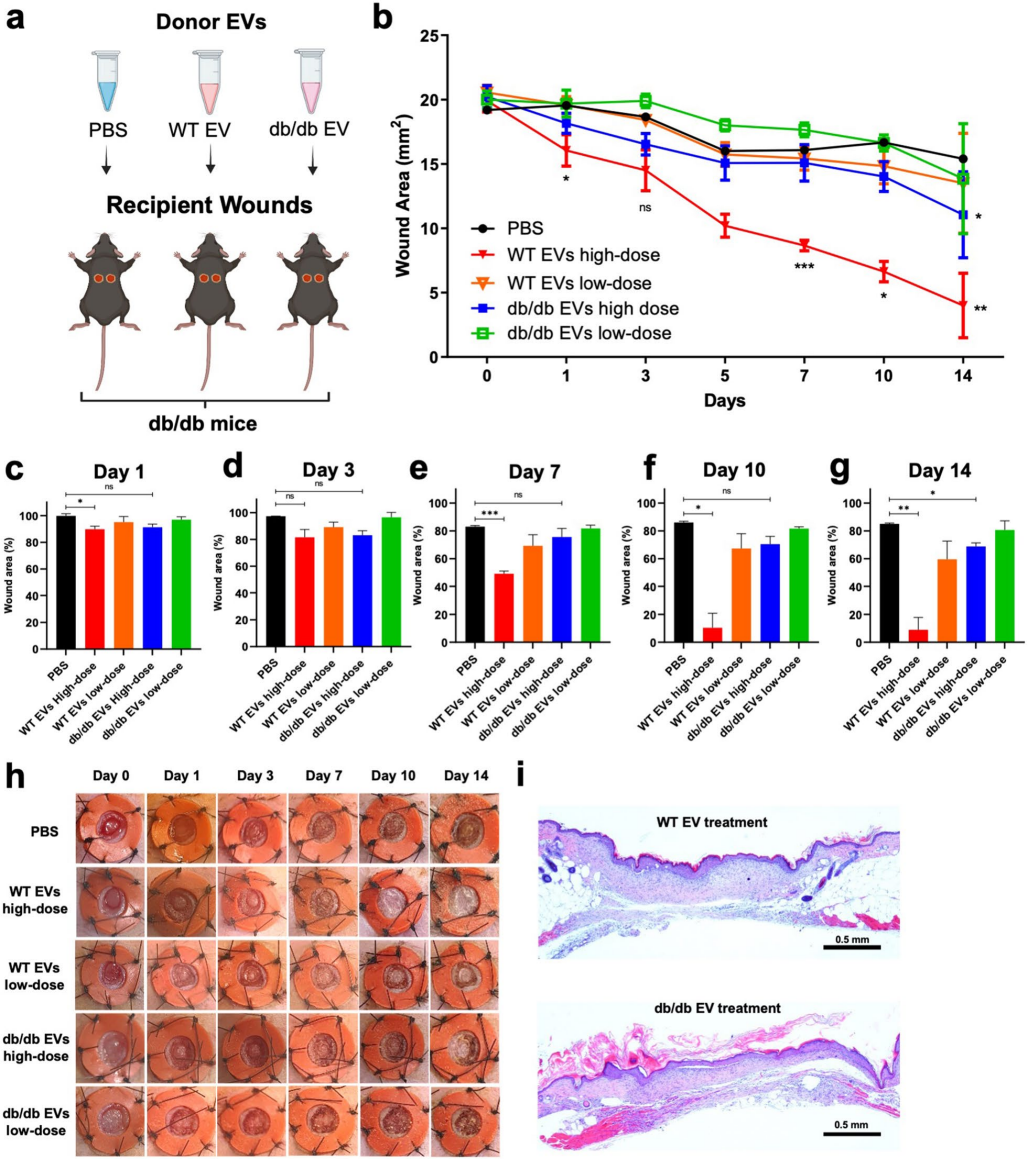
The pro-reparative phenotype of normal wildtype EVs is delayed in EVs from db/db mice. **a** Schematic of EV collection from sponge implants implanted in WT and db/db mice (i.e. Donor) followed by adoptive transfer to full thickness wounds into db/db mice (i.e. Recipient). **b** Kinetics of wound closure area following treatment with EVs enriched WT vs. db/db donor mice (2-way ANOVA of WT vs db/db high dose EV treated mice, *p*-value < 0.0001). Additional statistical analysis of wound closure efficiency is shown on **c** Day 1, **d** Day 3, **e** Day 7, **f** Day 10, and **g** Day 14 (Means + SD*, p*-value: *** < 0.001, ** < 0.005, * < 0.05). **h** Representative images of splinted wounds treated with EVs. **i** Representative hematoxylin and eosin staining of the injury site following treatment with WT vs. db/db EVs at 14 days (Scale bars, 0.5 mm)

To determine whether there were differences in the biological activity of WT vs. db/db EVs, we enriched EVs from PVA sponges implanted in WT or db/db mice, and adoptively transferred them into a full thickness cutaneous wound made in the dorsum of recipient db/db mice. EVs from each donor mouse genotype were administered once in either of two doses onto the wound sites of naïve db/db mice (Fig. 2a). Treatment of db/db wounds with donor EVs led to a dose-dependent acceleration of wound closure with WT EVs that contrasted with db/db EVs at the high EV dose (7.5 × 10^5^ particles/µL) (Fig. 2b). Statistically significant db/db-mediated uncoupling of the pro-reparative activity of EVs was observed at days 7–14 (Fig. 2c-g). Representative images of replicates (n = 4 at each time point for each condition) show delayed wound closure in db/db-treated EVs at the high dose (7 × 10^5^ particles/µL) (Fig. 2h) compared to treatment with WT EVs that was consistent with a delay in re-epithelialization of wounds treated with db/db EVs at day 14 (Fig. 2i, right).

### Identification of changes in EV protein payloads based on mass spectrometry

**Fig. 3:**
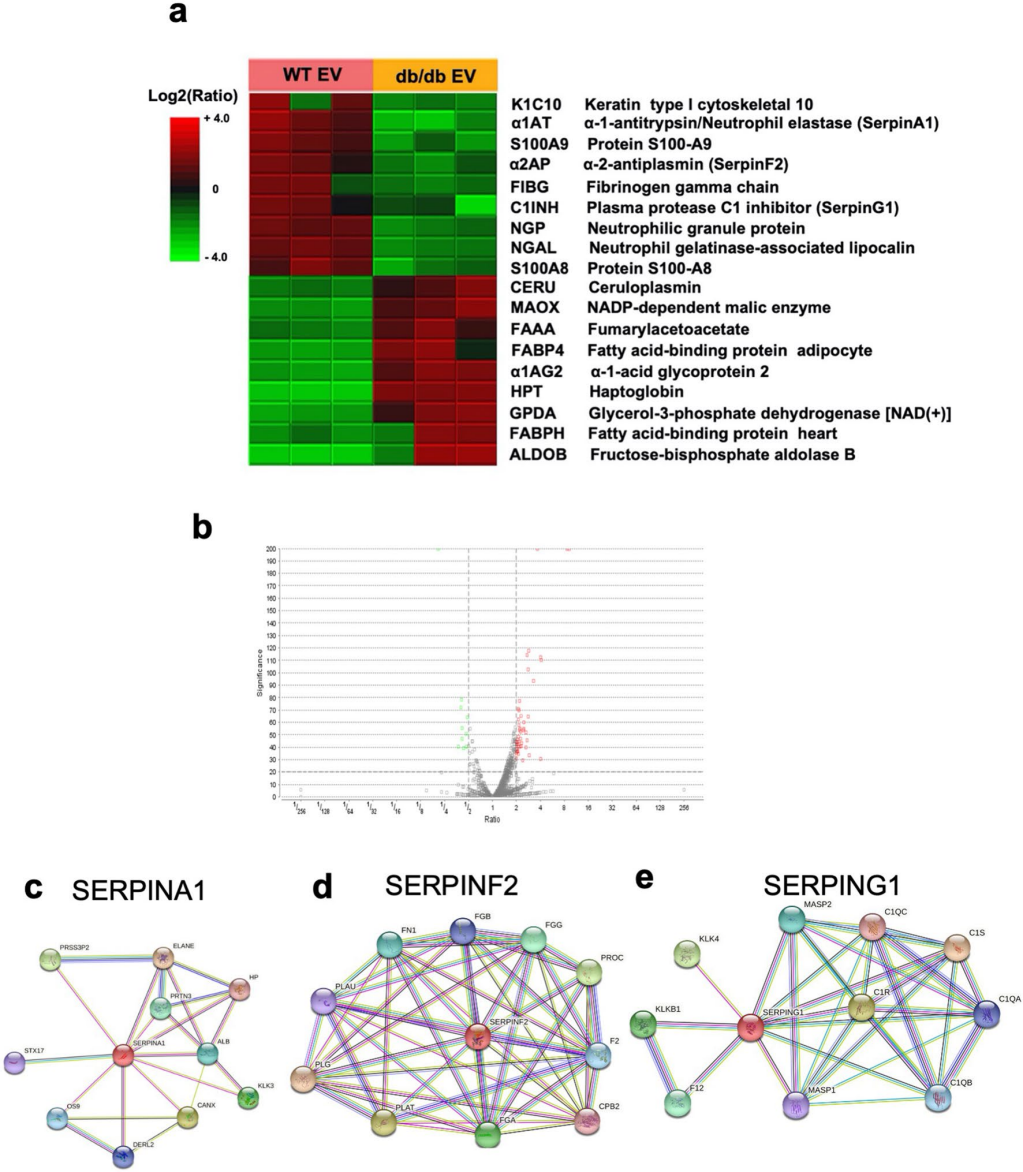
Proteomic analysis of EV protein payloads isolated from WT vs. db/db donors. **a** A heatmap of protein expression in WT and db/db EVs showing protein levels elevated in WT EVs compared to db/db EVs, and protein levels in WT EVs that are lower than db/db EVs. n = 3 independent animals of each genotype. **b** Volcano plot showing that the standard deviation of EV proteins identified and magnitude of fold-changes that support the high statistical significance of EV proteins identified. n = 3 biological replicates for each genotype. **c** String analysis of predicted proteins that may interact with SERPINA1 (red symbol), **d** SERPINF2 (red symbol), and **e** SERPING1 (red symbol). All representative images showed as observed in three independent experiments

Based on differences in biological activity between db/db and WT EVs in promoting wound closure, we subjected EVs from the db/db vs. WT donors to mass spectrometry to measure changes in EV proteins that could account for differences in their biological activity. We identified 5315 proteins (Additional file 2) that were differentially expressed in db/db vs. WT EVs, with a heat map showing changes in protein expression in biological replicates (n = 3 for each genotype) that were most highly down-regulated in db/db EVs (Fig. 3a, top) and proteins that were up-regulated in db/db EVs compared to WT EVs (Fig. 3a, bottom). Among the proteins that were down-regulated in the db/db EVs, three protease inhibitors α-1-antitrypsin 1 (α1AT), α-2-antiplasmin (α2AP), and plasma protease C1 inhibitor (C1INH) were identified as part of the Serpin family of Serine protease inhibitors and suggested their potential relevance in wound bed hemostasis, fibrinolysis, and inflammation. Additional factors relevant in neutrophil activation such as neutrophilic granule protein and neutrophil gelatinase-associated lipocalin were also decreased in db/db EVs. Of the proteins that were up regulated in db/db EVs, several proteins associated with metabolism were identified, consistent with the hypermetabolic phenotype of db/db mice. These proteomic analyses demonstrated that the EVs released in WT vs. db/db donor could be distinguished on the basis of the expression of protease inhibitors, neutrophil activation and metabolic enzymes. With a focus on examining a class of regulatory molecules relevant to the extracellular matrix, cell migration and re-epithelialization of the wound bed. A String analysis revealed linkages between SerpinA1 with STX17, KLK3 and OS9 (Fig. 3c), SerpinF2 with CPB2 and FGB (Fig. 3d), and SerpinG1 with KLK4, KLKB1, and C1R (Fig. 3e). Based on the relevance of Serpin activity in the wound bed, and EVs as nanocarriers of functional payloads, we examined the potential for over-expressing proteins identified as being down-regulated in a proteomics screen to then test their biological activity in keratinocyte migration and wound healing.

### Design and activity analysis of Serpin-loaded EVs in wound healing

**Fig. 4:**
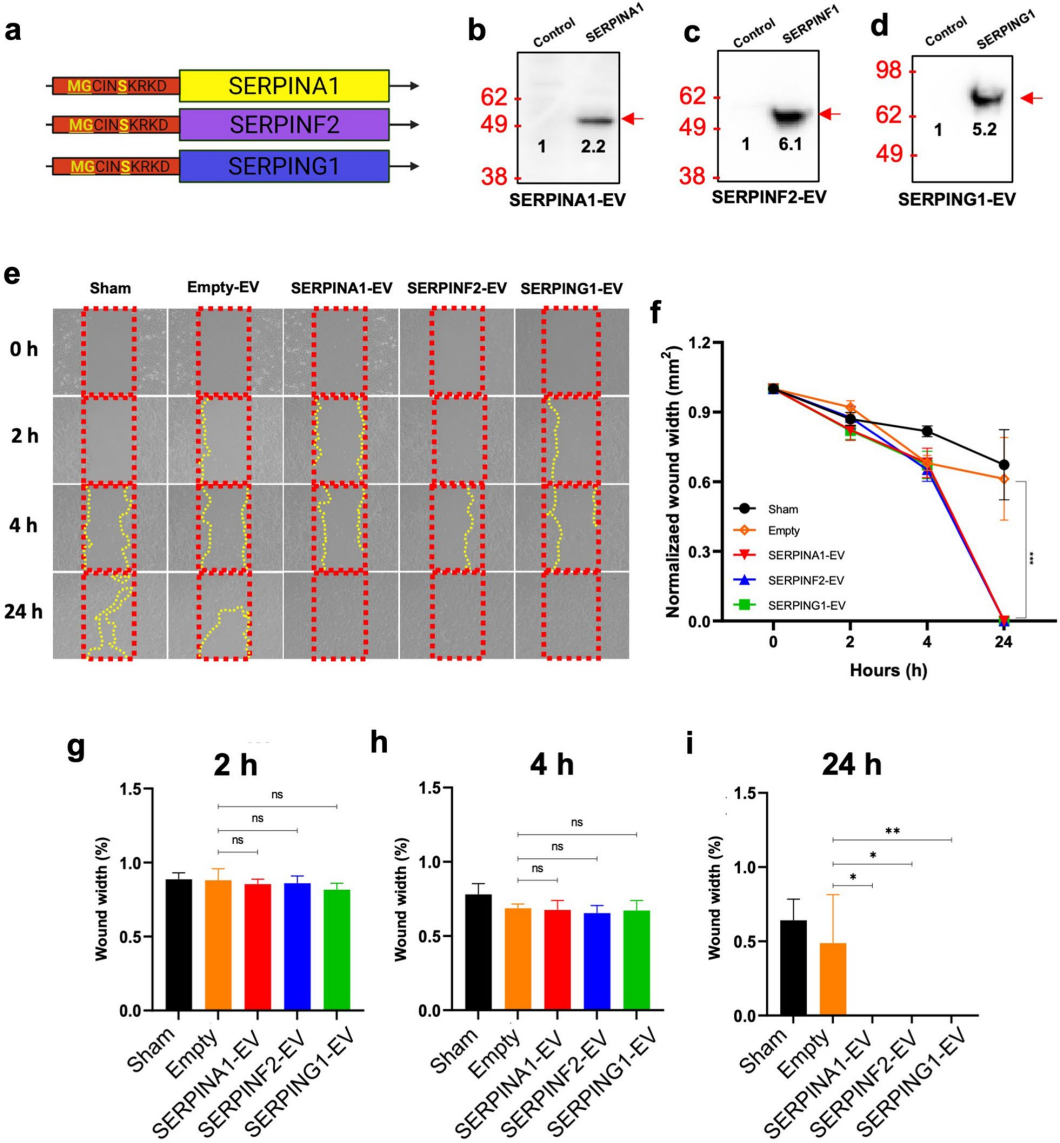
Testing of Serpin-loaded EV activity in vitro. **a** Fusions of an amino terminal myristoylation sequence with each Serpin were cloned into lentiviral vectors. **b-d** Validation of three Serpin-loaded EVs by immunoblotting in cultured media. Sham is PBS and Empty is the empty vector used for the myristoylation fusions. **e** Representative images of HaCaT cell closure kinetics. **f** Gap closure quantification of HaCaT cells treated with SERPINA1-EVs, SERPINF2-EVs, SERPING1-EVs, vs. empty vector, and sham (PBS). (*p*-value: *** < 0.001, n = 6). Statistical analysis of scratch assay on HaCaT cells by SERPIN-loaded EVs enriched from HEK293 donor cells on **g** 2 h, **h** 4 h, **i** 24 h (*p*-value: ** < 0.005, * < 0.05)

To determine the potential of engineering EV protein payloads as therapeutics in a wound healing model, we expressed proteins of interest as in frame fusions with an amino terminal sequence that incorporated a myristyolation sequence known to lead to the post-translational modification and preferential trafficking of modified proteins into EVs [30, 31]. Focusing first on the loading of a green fluorescent protein reporter expressed as a fusion protein (XP-GFP), we used lentivirus-mediated gene delivery to express XP-GFP in cultured HEK293 cells (Additional file 1: Fig. S5) from which EVs were enriched and subjected to a quantitative analysis to determine EV size, GFP fluorescence, tetraspanin CD81 expression, and uptake into HEK293 cells (Additional file 1: Fig. S6). These studies established that amino terminal fusions using myristoylation sequences localized proteins to EVs that were capable of delivering protein cargos to 30–50% of the cells treated with EVs as measured by GFP fluorescence. Based on these validations, we cloned each of the three Serpins identified as down-regulated in db/db EVs (Fig. 4a) as myristoylated fusion proteins to generate Serpin-loaded EVs (Additional file 1: Table. S2) for biochemical and activity testing. Densitometry analysis of immunoblots of EVs enriched from transduced HEK293 cells demonstrated Serpin expression in released EVs (Fig. 4b–d) compared to EVs from control parental cells. The activity of these Serpin-loaded EVs were then tested in an in vitro model of wound healing based on migration of human HaCaT keratinocyte cells [32–34]. Following removal of a dividing insert, HaCaT cells were treated once with each engineered EV type (1.20–1.7 × 10^5^ particles/µL), and changes in the cell gap quantified (Fig. 4e, f). The kinetics of closure following treatment with Serpin-loaded EVs was significantly accelerated at 24 h for each of the fusion proteins expressing Serpins (SERPINA1, SERPINF2, and SERPING1) compared to the empty vector control (Fig. 4g–i).

**Fig. 5:**
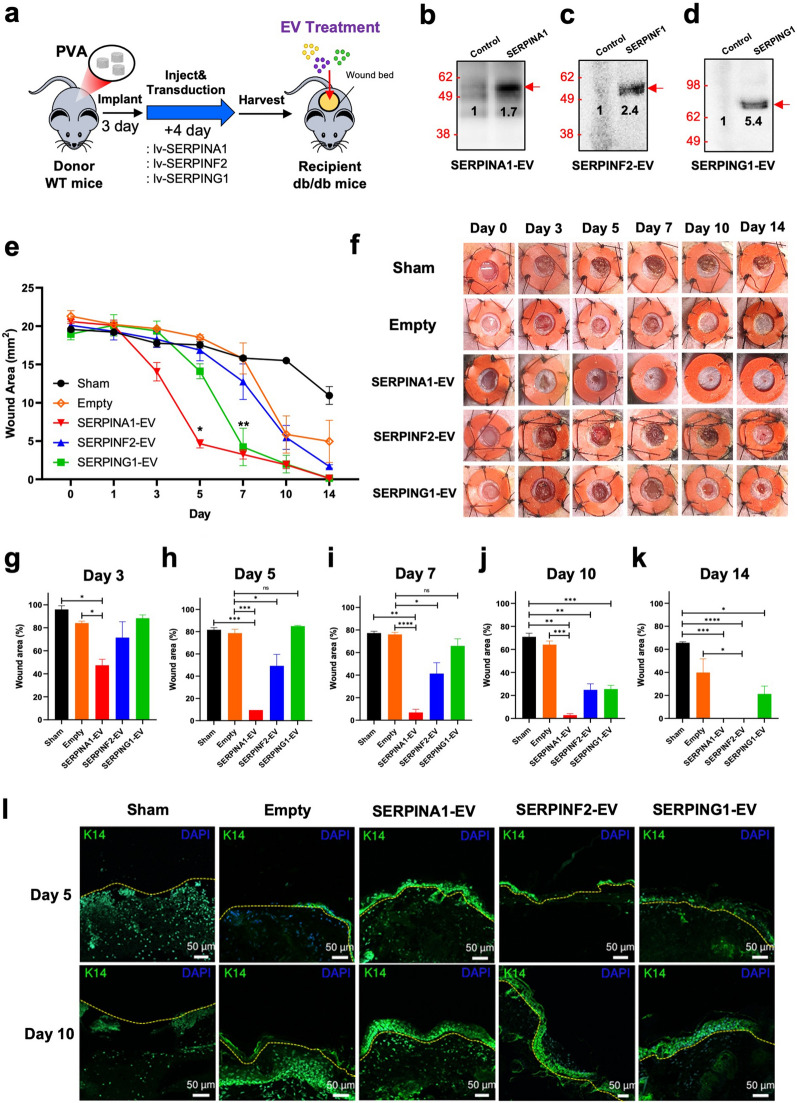
Testing of Serpin-loaded EV activity in vivo. **a** In vivo strategy to transduce cells infiltrating PVA sponge implants and enrich their EVs for assessment of wound closure activity following the expression, release and enrichment of EVs loaded with SERPINA1, SERPINF2, SERPING1, and empty vector controls. **b-d** Validation of Serpin expression in engineered EVs by immunoblotting EVs recovered from implants, with densitometric quantification shown on the blot. **e** Quantification of wound closure kinetics following adoptive transfer in the db/db mouse model of impaired wound healing. (2-way ANOVA, *p*-value < 0.0001 for SERPINA1 and SERPIN G1 vs. empty vector control, n = 6 in each arm) **f** Representative images of splinted wounds treated with Serpin-loaded EVs. Statical analysis of wound closure efficiency in vivo using SERPINA1-EVs, SERPINF2-EVs, and SERPING1-EVs on **g** Day 3, **h** Day 5, **i** Day 7, **j** Day 10, and **k** Day 14 (*p*-value:**** < 0.0001, *** < 0.001, ** < 0.005, * < 0.05). **l** Representative images of immunostaining at each time point to demonstrate expression of cytokeratin 14 at Day 5 and 10 post-injury. Statistical analysis of cytokeratin staining is shown in Fig. S7

To determine if Serpin-loaded EVs accelerated tissue repair in an in vivo model of impaired wound closure, we used a species-matched PVA sponge implant model (Fig. 5a) that combines in vivo gene transduction and EV harvest to test EV activity in vivo [6]. In this study PVA infiltrating cells were transduced in vivo with Serpin-expressing lentiviral vectors, and Serpin-loaded EVs were then harvested from the conditioned fluid of cells in the PVA implants. PVA cells were transduced with lentiviruses encoding fusions of SERPINA1, SERPINF2, SERPING1 with the same myristoylation tag used for the in vitro studies and compared with an empty vector control expressing only the tag. These EVs were enriched, counted, and the expression of the appropriate Serpin confirmed and quantified (Fig. 5b–d). To test the biological activity of the Serpin-loaded EVs, each was tested by adoptive transfer into the splinted full thickness wound model of impaired wound healing using db/db mice as the recipients. In contrast to kinetics of wound closure of PBS-treated (sham) db/db mice, wound closure was significantly accelerated by day 5 and 7 following a single dose treatment with EVs loaded with SERPINA1 and SERPING1, compared to empty vector control EVs (Fig. 5e) that was supported by representative images (Fig. 5f) and statistical analysis (Fig. 5g–k). To assess the effect of Serpin-loaded EVs on re-epithelialization we performed immunohistochemistry with an anti-cytokeratin 14 (K14) antibody and observed K14 staining within 5 days following treatment with SERPINA1 and SERPING1-loaded EVs compared to empty vector control (Fig. 5l). By Day 10, treatment with Serpins had increased immunostaining compared with sham and empty vector control EVs (Additional file 1: Fig. S7). Taken together these findings support the development of therapeutic EVs based on an EV loading strategy using fusion proteins and demonstrates the pro-reparative activity of SERPINA1 and SERPING1-loaded EVs in tissue repair.

## Discussion

**Fig. 6:**
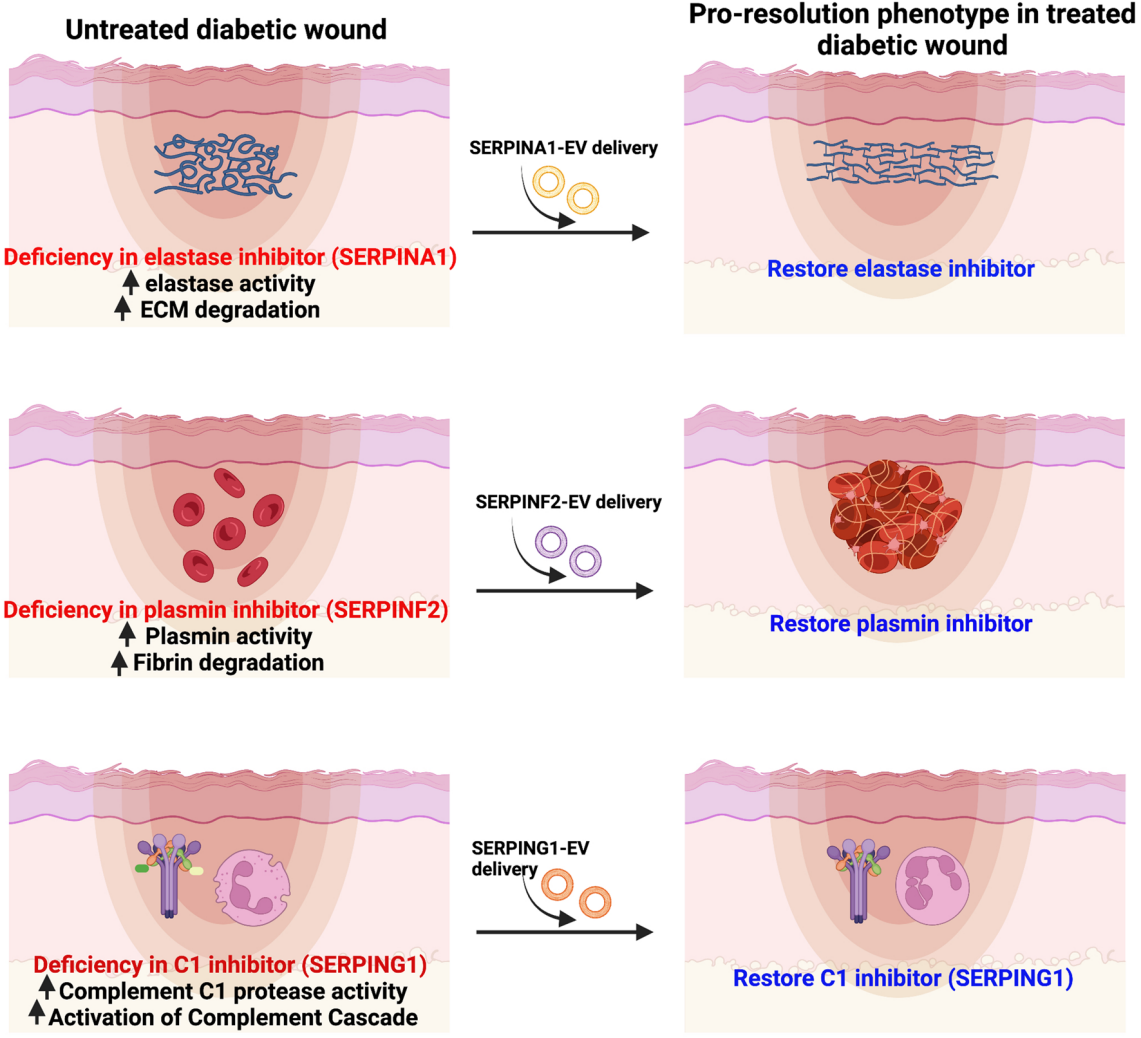
Model for Serpin-loaded EV action in accelerating a pro-resolution phenotype. (Top) In diabetic wounds, we propose that increased elastase activity increases degradation of the extracellular matrix (ECM) that can be reversed by delivery of SERPINA1-EVs to promote healthy ECM. (Middle) Decreases in plasmin inhibitor of diabetic wounds increases plasmin activity and degradation of fibrin that can be reversed by delivery of SERPINF2-EVs leading to formation of a beneficial fibrin scaffold. (Bottom) The loss of the inhibitor of the Complement C1 protease activity (C1) in diabetic mice that increases production of complement cascade products, which can be reversed by delivery of SERPING1-EVs that suppress the activation of complement cascade, activation of neutrophils, and promotes the resolution of the inflammation phase of wound repair. Created with Biorender.com

The application of EVs as therapeutics to promote tissue repair highlights their translational potential; however, the molecular mechanisms of EV action remain poorly understood. Questions remain regarding what factors mediate their efficacy, the identity of relevant payloads, and how EV biology is altered in a chronic vs. normal wound bed. We have previously shown that dysregulation of EV biogenesis pathways blocks wound repair [6] by affecting protein payload and immune cell recruitment relevant to the resolution of the inflammation phase wound repair. Here we focus on a genetically defined mouse model of Type 2 diabetes with impaired wound healing to assess EV biology in this process. We show that wildtype EVs accelerate wound closure compared to EVs from diabetic donors. Proteomic analysis of EVs isolated from diabetic mice have a deficit in the expression of a family of serine protease inhibitors, specifically Serpins A1 (anti-trypsin), Serpin F2 (anti-plasmin), and Serpin G1 (plasma protease C1 inhibitor). Given the role of Serpin A1 in inactivating elastases and Serpin F2 in regulating fibrinolysis by inactivating plasmin, and Serpin G1 in inhibiting the complement response [35], we propose that EVs are nanocarriers of key regulatory elements responsible for coordinating hemostasis, immune cell activation and the resolution phase of wound healing. We show that re-expression of the specific Serpins in EVs that are deficient in diabetic mice accelerated wound closure in an adoptive transfer strategy, and establishes the potential for Serpins to coordinate tissue repair (Fig. 6).

Dysfunction of immune function, fibrosis, and a non-migratory epidermis are all hallmarks of the impaired wound healing responses observed in diabetic patients, including diabetic foot ulcers, for which there are limited effective therapies [1]. For example, diabetic patients fail to activate key transcriptional networks in neutrophils and macrophages that are recapitulated in diabetic mouse models. These findings suggest that the lack of coordinated signaling and immune responses contribute to impaired re-epithelialization and delayed wound closure [36]. In these examples, biochemical mediators identified as down-regulated in the diabetic wound can be re-expressed to assess their potential to rescue tissue repair in chronic wounds as we have demonstrated here with Serpin-loaded EVs.

The multiple overlapping phases of hemostasis, inflammation, proliferation, and remodeling in wound healing are both multicellular and highly coordinated, suggesting that therapeutic approaches are needed that address defects in intercellular signaling between cells that are not otherwise in direct contact. The use of the PVA sponge to generate donor EVs takes advantage of the similar immune responses with the inflammation phase of wound healing and avoids the limitations of classic cell culture methods based on cultured single cell types on plastic in the presence of serum [37]. For the evaluation of EVs in the treatment of impaired cutaneous wound healing, normal EVs have been generally collected from cultured human cells [38], with recent studies showing that delivery of specific molecules affects wound closure in the diabetic model [14].

Based on evidence that fibrinolysis and changes in the extracellular matrix (ECM) scaffolding are essential to the coordinated resolution of the inflammation phase and re-epithelialization, our findings support a role for specific protease inhibitors in these processes [39, 40]. For example, protease inhibitors such as Serpins are critical molecular switches in ECM remodeling cascades that affect the recruitment of immune cells and re-epithelialization through a combination of direct contact and indirect intercellular signaling. Based on the pro-reparative potential of EVs in the diabetic wound [22, 41], our analysis of isolated wildtype and diabetic EVs has identified distinct protein payloads and biological activities in mediating wound repair. The impaired expression of protease inhibitors in diabetic wound EVs underscores the importance of specific proteases that mediate remodeling of the ECM, drive the recruitment and activation of immune cells in wound closure, and are indicative of a more general dysfunction of proteases in diabetic wounds.

For example, Serpin A1 encodes the alpha1-proteinase inhibitor (α1PI), which acts upon trypsin as well as neutrophil elastases [42]. Diabetic wounds have increased elastase associated with infection and worsening of ulcers [43], consistent with our observation of decreased levels of the SerpinA1/α1PI elastase inhibitor in diabetic wound EVs. While the mechanisms of EV-mediated SerpinA1 have yet to be elucidated, SerpinA1/α1PI also has non-enzymatic activity in its ability to bind to the cell surface and promote migration [29, 42]. Surface binding of EVs, especially at the leading edge where membrane-bound EVs loaded with ECM-remodeling protease/protease inhibitors, can enhance cell migration by modifying intracellular actin re-organization that promotes directional cell adhesion [44]. Other examples of the pathogenesis of reduced SerpinA1/α1PI levels include increased neutrophil elastase in the bloodstream leading to acute inflammation, degradation of lung elasticity, and liver cirrhosis [42].

The decreased levels of Serpin F2/antiplasmin observed in EVs in the diabetic wound support the role for factors regulating coagulation and fibrinolysis in wound healing [45]. In wound healing, mice lacking plasminogen, the zymogen form of plasmin, tissue plasminogen activator or urokinase plasminogen activator all have impaired wound healing responses [46, 47]. While free Serpin F2/antiplasmin inhibits plasmin formation, the fibrin-bound form regulates clot lysis [45]. Studies of mice lacking Serpin F2/antiplasmin show accelerated angiogenesis and wound closure [48] further supporting a role for the fibrinolytic cascade in regulating wound closure kinetics, although the timing and localization of specific targets remain poorly understood. Therefore, wound healing disorders may respond to increases in protease inhibitors such as Serpin F2/antiplasmin that accelerate fibrinolysis and promote conversion of a wound microenvironment from pro-inflammatory to pro-resolution and increased wound closure [49].

Serpin G1/C1-inhibitor is an acute-phase protein that increases in the circulation in response to injury, and functions to inhibit the complement system. Deficiency in Serpin G1/C1-inhibitor also permits kallikrein activation, and the production of the vasoactive peptide bradykinin [50]. In the wound bed, administration of a Serpin G1/C1-inhibitor reduces local inflammation and capillary leakage, although the acceleration of wound closure may depend on the duration of Serpin G1/C1-inhibitor administration [51, 52]. With Serpins often classified as acute phase proteins that regulate inflammation, expressing specific Serpins in EVs may promote wound closure by delivering enzyme inhibitors in EVs that have an EV-dependent tropism for specific microenvironments of the wound bed. For example, elastase levels in diabetic ulcer tissue were significantly higher in wounds with infections and presented with a delayed wound healing profile [53]. Moreover, EVs delivering enzymes that regulate the remodeling of ECM can localize protease inhibitors at the interface of plasma membrane and release protein payloads at membrane domains regulating the leading edge of migrating cells [54].

The rationale for the delivery of EVs loaded with specific protein payloads is based on the identification of specific Serpins that are down-regulated in the diabetic EVs, which can then be tested for their capacity to restore wound closure kinetics of a wild type full thickness wound. Our strategy uses lentiviral constructs to express a target protein as a fusion with a membrane domain that traffics proteins to EVs and anchors them to the membrane [30, 55]. Membrane domains that mediate the trafficking of protein payloads into EVs based on a myristoylation domain have similarities with viral budding that can be exploited for delivery of custom EV protein payloads [56–58].

EV-based therapies have emerged as a significant area of interest in the treatment of ischemia, infection, impaired wound healing, and cancer, however, none are FDA-approved to date. The over-arching concept behind the development of EV-based therapeutics is their potential as stable delivery vehicles of payloads that regulate intercellular signaling, especially when compared to small molecules in solution or cell therapy [59]. EVs are complex in terms of the range of their protein and nucleic acid payloads, and heterogenous in terms of the different populations of EVs present in a multi-cellular microenvironment where a given cell type may release different EVs depending on its metabolism and activation state [6]. As in our previous EV proteomic studies [6, 60], we use proteomics to identify proteins that are differentially expressed in EVs which in this study we show can be rescued using a re-expression system. The use of a species and strain-matched donors for the collection EVs for the analysis of the effect of the diabetic host on their EV payload is essential for the determination of their ability to promote wound closure following adoptive transfer into a full thickness chronic wound.

## Conclusions

The discovery of a deficiency of Serpin expression in the EVs of diabetic mice led to the hypothesis that over-expression of such Serpins in engineered EVs could be used to rescue impaired wound healing. The combination of target discovery leading to the development of Serpin-loaded EVs established the biological activity of engineered Serpins with applications that may well extend beyond the molecular targets and injury models tested here.

## Methods

### Mice, PVA sponge implants and wound healing assays

All animal experiment were conducted in accordance with the protocols approved by the Institutional Animal Care and Use Committee of the University of California, San Diego. Male 10–16-week-old C57BL/6J mice (JAX #0664) and Leprdb/db mice (JAX #0697) were maintained on a 12 h light/dark cycle. Subcutaneous implants of sterile polyvinyl alcohol (PVA) sponges (PVA unlimited, Warsaw, IN, USA) inserted (n = 3 per site) in dorsum of each animal and the surgical site closed with nylon monofilament suture (# MV-663-V-19 mm, Oasis, IL, USA) [25, 61]. Animals recovered in the presence of sufficient food and water supply. Mice bearing PVA sponges were incubated as indicated for up to 14 days, the sponges removed under anesthesia, transferred into 500 µl of PBS in a microtube, and the sponges briefly compressed with a forceps 3–4 times to release cells and EVs from the sponge. The sponges were then removed, and cells separated from the PVA fluid by centrifugation at 300 × g for 5 min for analysis by flow cytometry and quantitative PCR, while EVs were enriched from the supernatant for in vitro testing as described below and tested in adoptive transfer assays into full thickness wounds in naïve db/db mice. For these assessments of EV activity upon the kinetics of wound healing assay in the db/db model, hair was removed, a full thickness 4 mm punch made (#P450, Acuderm inc., FL, USA), the site splinted with silicone ring (Thickness: 0.5 mm, outside diameter: 12 mm, inside diameter: 6 mm) (#33,350,174, MCS, Mableton, GA, USA) and the ring immobilized with nylon suture as previously described [18, 62] were. EVs were added to the wound site in 10–50 µL, covered with Tegaderm (#1622w, 3 M, Maplewood, MN, USA), and the wound site imaged daily with a digital camera (Galaxy 10e, 1200 pixels, AF, F1.5/F2.4 super speed dual pixel, Samsung, Seoul, Korea) and analyzed by Image J (1.53e version, National Institutes of Health, Bethesda, USA). Hematoxylin and eosin-stained tissue sections of skin were prepared from formaldehyde fixed paraffin embedded, and cryosections prepared in Tissue-Tek® OCT compound (Cat#4583, Sakura® Finetek, Torrance, CA, USA), stained with Cytokeratin 14 antibodies (Cat#10,143-1-AP, Proteintech, Rosemont, IL, USA) and imaged with a laser scanning confocal microscope (ECLIPSE Ti2, Nikon Instruments Inc. Melville, NY, USA). These EV assays were used for the assessment of WT vs db/db EV activity as well as for the analysis of EV engineered to express specific Serpins as described below. In both studies, EVs were prepared from mice bearing PVA sponge implants, enriched, quantified and then adoptively transferred to full thickness splinted wounds as described above.

### Flow cytometry

For the analysis of cells recruited to the PVA sponge model used for the harvest of EVs, cells were subjected to flow cytometry using Fc block (Cat#130-092-575, Miltenyi Biotec, San Diego, CA, USA), followed by staining with antibodies specific for the following immune cell markers from Miltenyi Biotec. (CD11b, #130-109-287; CD11c, #130-110-840; CD45, #130-110-803; CD44, #130-119-127; F4/80, #130-102-422; Gr1, #130-102-233; Ly6G, #130-107-912, Ly6C, #130-123-796, MHCII, # 130-119-122; CD4, #130-118-696, and CD3, #130-117-788). Propidium iodide (#130-093-233, Miltenyi Biotec, San Diego, CA, USA) was used to exclude dead cells. Isotype antibodies were used for all fluorescence studies. (VioBlue/PacBlue, #130-113-454; VioGreen/BV510, #130-113-456; FITC, #130-113-449; PE, #130-113-450; PE-Vio770, #130-113-452; APC, #130-113-446; and APC-Vio770/Fire750, #130-113-447, Miltenyi Biotec, CA, USA). All flow cytometry was performed on a MACSQuant 10 instrument (Miltenyi Biotec, San Diego, CA, USA) and analyzed using FlowJo software (Version 10.7.1, Becton, Dickinson and Company, Franklin Lakes, NJ, USA).

### Isolation of EVs

EV isolation from PVA sponge implants was based on density gradient ultracentrifuges as previously described [4, 63, 64], with an initial spin of 10,000×*g* for 30 min at 4 °C to separate EVs from the PVA fluid. Opti prep gradient (#D1556, Sigma Aldrich, CA, USA), was prepared as 8% (1.068 g/ml), 10% (1.078 g/ml), 14% (1.098 g/ml), 20% (1.127 g/ml), 26% (1.156 g/ml), and 30% (1.175 g/ml) solutions layers overlaid with supernatant in polycarbonate ultracentrifuge tube (#343,778, Beckman coulter, CA, USA) (Rotor #TS55, k factor: 50 k, Beckman coulter), and fractionated at 259,000 × g (Accel:4/Decel: 9) for 2 h in a Beckman Optima Max-XP Ultracentrifuge. After centrifugation, 10 × 100 µl fractions were collected, protein concentration determined by BCA assay (#23,227, Thermo Fisher Scientific, Waltham, MA, USA). For assays of protein expression, EVs were solubilized in RIPA lysis buffer (#89,901, Thermo Fisher Scientific), while for quantification of EV size, concentration, mass spectrometry, vFC or biological activity, EVs from control and experimental groups were normalized based on concentration as indicated.

EVs isolated from cultured media for in vitro assays was performed using Exoquick kit (#EQULTRA-20A-1, SBI, CA, USA) and following manufacturer’s recommendations. Briefly, cell culture medium was centrifuged at 3000×*g* for 15 min to remove cell debris, supernatant transferred to a new tube and incubated overnight at 4 ℃ with Exoquick. The Exoquick/media mixture was centrifuged at 3000×*g* for 10 min, the supernatant aspirated, and the pellet resuspended in PBS for subsequent concentration, sizing, immunoblotting and biological activity studies.

### Quantification of EVs and concentration and size distribution by vesicle flow cytometry

EV samples diluted by PBS, and stained with a fluorogenic membrane stain (vFRed, Cellarcus Biosciences), a cytoplasmic stain (CFSE, Cellarcus) and EV surface markers in a total volume of 50 µl in a 96 well v bottom plate for 1 h at ambient temperature, according to manufacturer’s instructions. The optimal concentrations of antibody and other reagents was determined by the manufacturer via titration and provided at 10 × the final staining concentration. Stained samples were diluted 1000-fold in vesicle staining buffer and analyzed on the flow cytometer. The dilutions series protocol determines the EV concentration, assay dynamic range, and the optimal dilution for subsequent cargo analysis.

### Transmission electron microscopy

For the imaging of EVs by transmission electron microscopy (TEM) on grids, the PELCO easiGlow system (91000S, Ted Pella, Inc) was using for hydrophilization onto grids (Cat# 01,754-F, Formvar, 200 mesh, copper, Ted Pella, Inc., Redding, CA, USA). Grids were washed and stained with uranyl acetate, and imaged with a Jeol 1400 plus TEM at 80 keV (Jeol USA, Peabody, MA, USA).

### Sample preparation and LC-Mass Spectrometry

EVs samples from PVA implants were isolated by density ultracentrifugation as described above and analyzed in the Biomolecular and Proteomics Mass Spectrometry Facility at UCSD. For each sample, guanidine-HCl was added to each sample to final concentration of 6 M, boiled for 10 min and cooled at room temperature for 5 min, with this cycle repeated three times. Following methanol precipitation and removal of the supernatant, the pellet was suspended in 8 M Urea in 100 mM (Tris pH 8.0). Samples were brought to a final concentration of 10 mM TCEP (2-carboxyethyl phosphine) and 40 mM Chloro-acetamide solution. Three volumes of 50 mM Tris pH 8.0 were added to the sample to reduce the final urea concentration to 2 M. Trypsin was add (1:50 ratio), incubated at 37 ℃ for 12 h, samples acidified using TFA (0.5% TFA final concentration) and desalted using C18-StageTips (#87,782, Thermo Fisher) as described by the manufacturer protocol. The peptide concentration of sample was measured using BCA after resuspension in TMT buffer. For high pH fractionation, the Pierce ™ High pH Reversed Phase Peptide Fractionation Kit (#84,868, Thermo Fisher) to generate 8 unique peptide fractions that were analyzed by ultra-high-pressure liquid chromatography (UPLC, Thermo Dionex UltiMate™ 3000 RSLC nano System) (#ULTIM3000RSLCNANO, Thermo Fisher) coupled with tandem mass spectroscopy (LC-MS/MS) using nano spray ionization. The nano-spray ionization experiments were performed using an Orbitrap fusion Lumos hybrid mass spectrometer (Model#IQLAAEGAAPFADBMBHQ, Thermo Fisher) interfaced with nanoscale reversed-phase UPLC using a 25 cm, 75-micron ID glass capillary packed with 1.7-µm C18 (130) BEHTM beads (Waters corporation). Peptides were eluted from the C18 column into the mass spectrometer using a linear gradient (5–80%) of ACN (Acetonitrile) at a flow rate of 375 μl/min for 120 min. The buffers used to create the ACN gradient were: Buffer A (98% H2O, 2% ACN, 0.1% formic acid) and Buffer B (100% ACN, 0.1% formic acid). Mass spectrometer parameters are as follows; an MS1 survey scan using the orbitrap detector (mass range (m/z): 400–1500 (using quadrupole isolation), 60,000 resolution setting, spray voltage of 2200 V, Ion transfer tube temperature of 290 ℃, AGC target of 400,000, and maximum injection time of 50 ms) was followed by data dependent scans (top speed for most intense ions, with charge state set to only include + 2–5 ions, and 5 s exclusion time, while selecting ions with minimal intensities of 50,000 at in which the collision event was carried out in the high energy collision cell (HCD Collision Energy of 38%) and the first quadrupole isolation window was set at 0.8 (m/z). The fragment masses were analyzed in the orbitrap detector (mass range (m/z) by automatic scan with first scan at m/z = 100. The resolution was set at 30,000 resolutions. The AGC Target set to 30,000, and maximum injection time was 54 m-sec. Protein identification and quantification was carried out using Peaks Studio 8.5 (Bioinformatics solutions Inc., Canada).

### Western blot

All EVs subjected to immunoblotting were quantified by BCA assay kit (#23,225, Thermo Fisher), samples prepared in NuPAGE™ LDS Sample Buffer (#NP0008, Thermo Fisher), separated using the 12% Bis-Tris Mini Gel (#NP0342BOX, Thermo Fisher), transferred to PVDF membrane (#LC2005, 0.45 µm, 8.3 × 7.3 cm, Thermo Fisher), and blocked with 3% Nonfat Dry Milk Cell (NFDM) (Cat# 9999, CST, MA, USA) in 1X Tris-buffered saline (#9997, CST, MA, USA) with 0.05% Tween 20. Primary antibodies used were CD81 (#10,037, CST), CD63 (#PA5-92,370, Invitrogen, USA), CD9 (#PA-5–85,955, Invitrogen), Alix (#92,880, CST), SERPINA1 (#TA500374s, Origene, USA), SERPINF2 (#PA5-81,014, Thermo Fisher), and SERPING1 (#PA5-81,015, Thermo Fisher) at a 1/1000 dilution. Anti-rabbit IgG, HRP-linked (#7074, CST) or anti-mouse IgG, HRP-linked antibodies at 1/1000 dilution (Cat# 7076, CST, USA) were used as secondary antibodies, and blot incubated with Pierce™ ECL western blotting substrate reagent (#32,209, Thermo Fisher), blots imaged with a Xenogen IVIS-Lumina (Caliper Life Sciences Inc., Hopkinton, MA, USA) and the band intensities quantified using Living Image software (Ver.4.3.1, Caliper Life Sciences).

### Cloning and Lentivirus production

Lenti-X 293 T cells (#632,180, TakaraBio) were used for the production of lentivirus based on Lenti-XPack vectors (System Biosciences, Palo Alto, CA) that contained the EV signal peptides as an N-terminal fusion with a multiple cloning site (pLenti-XPack-MCS (#XPAK710PA-1) or as fusion with GFP (pLenti-XPack-GFP, #XPAK510PA-1). Primer design tools from TAKARA (https://www.takarabio.com/learning-centers/cloning/primer-design-and-other-tools) were used to amplify SERPIN genes from cDNAs (Origene) encoding human SERPINA1 (#RC202082), SERPINF2 (#RC228342) or SERPING1 (#RC203767) flanked by Xho I and NotI restriction enzyme sites for cloning into pLenti-XPack-MCS vector. The following primers were used for SERPINA1-F (5′- GCA AAG ATG CCT CGA GGA TGC CGT CTT CTG TCT CGT G -3’) and SERPINA1-R (5′- AGA ATT CTC GCG GCC GCT TAT TTT TGG GTG GGA TTC ACC AC -3′); SERPINF2-F (5′- GCA AAG ATG CCT CGA GGA TGG CGC TGC TCT GGG G -3′) and SERPINF2-R (5′- AGA ATT CTC GCG GCC GCT CAC TTG GGG CTG CCA AAC TGG -3′) and SERPING1-F (5′- GCA AAG ATG CCT CGA GGA TGG CCT CCA GGC TGA CC -3’) and-SERPING1-R (5′- AGA ATT CTC GCG GCC GCT CAG GCC CTG GGG TCA TAT ACT CG -3′). PCR fragments were linearized and cloned using the In-fusion kit (All In-fusion mix Plus, #638,917, TAKARA). The Lenti-vpak packaging kit (#TR30037, OriGene Technologies Inc, Rockville, MD, USA) was used for virus production, with lentivirus being collected and concentrated from conditioned media using the Lenti concentrator (#TR30026, OriGene Technologies Inc) and quantified using Lenti-X GoStix Plus (#631,280, TaKaRa Bio USA Inc, San Jose, CA, USA) that measures the expression of lentiviral p24 protein using GoStix Value software (Takara). Lentiviral stocks of matched titer were used for the subsequent transduction of either human HEK293T cells from which EVs would be collected for the treatment of HaCaT cells. PVA sponges were implanted 3 days prior to injection of the lentivirus into the sponge implants to facilitate in vivo transduction of infiltrated leukocytes. After an additional 4 days to allow for gene expression, cells and fluid from the PVA implant (Additional file 2) were harvested with EVs enriched from the fluid and adoptively transferred to full thickness wounds to assess activity by wound closure analysis as described above.

### Serpin-loaded EV activity cell migration assay

HEK293T (CRL-1573, ATCC, Bethesda, MD) cells were transduced with lentivirus (10,000 to 15,000 particles), incubated for 48 h in serum complete media, cells then washed with PBS and the media replaced with a serum-free medium for an additional 24 h. From this serum free media, EVs were concentrated, quantified, and immunoblotted as described above prior to testing in a migration assay using HaCaT cells cultured on 2-well dishes (#81,176, Ibidi, Gräfelfing, Germany). After 24 h seeding of cells, an insert was removed, the cell culture media replaced with EV containing media as described, cells imaged over 24 h using a CCD camera (Retiga R6, Teledyne photometrics, Tucson, AZ, USA) on an Olympus IX70 microscope to measure changes in cell migration. All images analyzed using OCULAR v1.0.3.110 software and Image J.

### Statistical analysis

All statistical analyses were performed with Prism 6.0 (Graph pad Software, La Jolla, CA, USA). Descriptive results of continuous variables were expressed as the mean ± standard deviation (SD) for normally distributed variables. Differences between different groups were compared by ANOVA for analysis of two or more groups in the kinetic studies, and Student’s t-test for pairwise comparisons with p-values indicated as **** < 0.0001, *** < 0.001, ** < 0.005, * < 0.05 considered to be statistically significant. All statistical analyzes and representative images are presented as observed in at least 3 independent experiments.

## Supplementary Information

**Additional file 1: Figure S1.** Statistical analysis of CD marker expression in PVA infiltrates at various time points. In support of the piechart analyses in Fig. 1, immune cells infiltrating PVA sponges were quantified at each time point focusing on: a Macrophages, b Inflammatory monocytes, c Neutrophils, d Dendritic cells, and e T cells (*p*-value: ***<0.001, **<0.005, *<0.05). **Figure S2.** Evaluation of EV biogenesis related gene expression in the PVA cells by qRT-PCR depending on the 2, 7, and 14 days. Measurements of changes in gene expression normalized to GAPDH in cells infiltrating PVA sponges focusing on the following canonical biogeneis genes: a Rab5a, b Rab5b, c Rab27, d Rab27b e Rab11a, f VPS4a, g TSG101, and h Rab35 (p-value: **<0.005, *<0.05). **Figure S3.** Analysis of baseline cell infiltrate in PVA sponges of WT vs. db/db mice, from which EVs were harvested. Flow cytometry analysis of immune cells recruited to the PVA sponge at 14 days post-implantation focusing on : a monoyctes based on CD11b^+^Ly6C^high^ cells as a subset of CD45^+^ cells, b neutrophils based on Ly6G^+^Ly6C^low^ cells as a subset of CD45^+^ cells, c macrophages based on CD11b^+^F4/80^+^ cells as a subset of CD45^+^ cells, d DCs based on MHCII^+^ cells as a subest of CD11b^+^CD11c^+^ cells, e T cells based on CD4^+^ cells as a subset of CD3^+^ cells, f CD8^+^ T cells as a subset of CD3^+^ cells, and g Regulatory T cells based on CD4^+^CD25^+^ as a subset of CD3^+^ cells. (*p*-value: *<0.05). **Figure S4. **Validation of EVs derived from WT and db/db mice by vFRed analysis. a Size distribution of WT EVs. b Plot diameter data of WT EVs. c Expression of Annexin V-PE on WT EVs. d Expression of tetraspanins CD9, CD63 and CD81 (TS-PE)on WT EVs. e Histogram of Annexin V-PE expression on WT EVs normalized to buffer only (grey filled in). f Histogram of TS-PE on WT EVs normalized to buffer only (grey filled in). g Size distribution of db/db EVs. h Plot diameter data of db/db EVs. i Expression of Annexin V-PE on db/db EVs. j Expression of TS-PE on db/db EVs. k Histogram of Annexin V-PE expression on db/db EVs normalized to buffer only (grey filled in). l Histogram of TS-PE on db/db EVs normalized to buffer only (grey filled in). **Figure S5. **Validation of lentivirus titer testing using HEK293 cells. a Representative comparison of lentivirus yields b based on titer testing of lentiviral p24 protein using GoStix analysis per manufacturers recommendations.** Figure S6. **A representative example of the analysis of EVs engineered to express specific proteins. a EVs collected from the conditioned media of parental cells as a negative control (Left, naïve HEK293 donor cells) and enriched by density ultracentrifugation were subjected to vFRED staining to determine EV diameter as described in the Materials and Methods. GFP-loaded EVs (XP-GFP) in absence (Left) or presence (Right) of anti-CD81-PE tetraspanin were analyzed for b GFP fluorescence and c expression of CD81. d and e An overview and quantification of GFP-EV internalization into HEK293 cells (filled in) compared to buffer control (open). **Figure S7.** Quantification of K14 immunohistochemistry. The fluorescent intensity of ant-K14 stained tissue sections from Figure 5l were quantified using Image J. A minimum of 3 fields were analyzed for each of the EV treated samples (p-value: * <0.05).** Table S1. **Detail information of primer for extracellular vesicle biogenesis related genes.** Table S2. **Primer sequences for cloning of XP tag in-frame with SERPINA1, SERPINF2, and SERPING1.

**Additional File 2:** Proteomic data from the Mass Spectrometry analysis of proteins detected in EVs from wild-type and diabetic db/db mice.

## Data Availability

Data supporting the primary findings are included in the main figures, the Supplementary Data, with additional data regarding EV characterization available upon request.

## Abbreviations

EV: Extracellular vesicles
Serpin: serine protease inhibitor
α1AT: Alpha-1-proteinase inhibitor
α2AP: α-2-Antiplasmin
C1INH: Plasma protease C1 inhibitor
ECM: Extracellular matrix
PVA: Polyvinyl alcohol
WT: Wild type
GFP: Green fluorescent protein
