# Supplementary material for: Serpin-loaded extracellular vesicles promote tissue repair in a mouse model of impaired wound healing
Source: J Nanobiotechnology. 2022 Nov 5;20:474. doi: 10.1186/s12951-022-01656-7 (PMC9636779; doi:10.1186/s12951-022-01656-7)
Supplement: Supplementary file 1 — Additional file 1: Figure S1. Statistical analysis of CD marker expression in PVA infiltrates at various time points. In support of the piechart analyses in Fig. 1, immune cells infiltrating PVA sponges were quantified at each time point focusing on: a Macrophages, b Inflammatory monocytes, c Neutrophils, d Dendritic cells, and e T cells (p-value: ***<0.001, **<0.005, *<0.05). Figure S2. Evaluation of EV biogenesis related gene expression in the PVA cells by qRT-PCR depending on the 2, 7, and 14 days. Measurements of changes in gene expression normalized to GAPDH in cells infiltrating PVA sponges focusing on the following canonical biogeneis genes: a Rab5a, b Rab5b, c Rab27, d Rab27b e Rab11a, f VPS4a, g TSG101, and h Rab35 (p-value: **<0.005, *<0.05). Figure S3. Analysis of baseline cell infiltrate in PVA sponges of WT vs. db/db mice, from which EVs were harvested. Flow cytometry analysis of immune cells recruited to the PVA sponge at 14 days post-implantation focusing on : a monoyctes based on CD11b+Ly6Chigh cells as a subset of CD45+ cells, b neutrophils based on Ly6G+Ly6Clow cells as a subset of CD45+ cells, c macrophages based on CD11b+F4/80+ cells as a subset of CD45+ cells, d DCs based on MHCII+ cells as a subest of CD11b+CD11c+ cells, e T cells based on CD4+ cells as a subset of CD3+ cells, f CD8+ T cells as a subset of CD3+ cells, and g Regulatory T cells based on CD4+CD25+ as a subset of CD3+ cells. (p-value: *<0.05). Figure S4. Validation of EVs derived from WT and db/db mice by vFRed analysis. a Size distribution of WT EVs. b Plot diameter data of WT EVs. c Expression of Annexin V-PE on WT EVs. d Expression of tetraspanins CD9, CD63 and CD81 (TS-PE)on WT EVs. e Histogram of Annexin V-PE expression on WT EVs normalized to buffer only (grey filled in). f Histogram of TS-PE on WT EVs normalized to buffer only (grey filled in). g Size distribution of db/db EVs. h Plot diameter data of db/db EVs. i Expression of Annexin V-PE on db/db EVs. j Expression of TS-P [file 12951_2022_1656_MOESM1_ESM.docx]

**Additional file 1**

**Serpin-loaded extracellular vesicles promote tissue repair**

**in a mouse model of impaired wound healing**

Dong Jun Park^1^, Erika Duggan^3^, Kayla Ho^1^, Robert Dorschner^2^, Marek Dobke^1^,
John Nolan^3^, and Brian P. Eliceiri^1,*^

^1^Departments of Surgery and ^2^Dermatology, University of California San Diego, 9500 Gilman Drive, MC 8236, La Jolla, CA 92093-8236, USA

^3^Scintillon Institute, 6868 Nancy Ridge, San Diego, CA 92121, USA

*Correspondence:

**Brian P. Eliceiri, PhD**

Department of Surgery, University of California San Diego, 9500 Gilman Drive, MC 8236, San Diego, CA 92093-8236, USA

Email: [beliceiri@health.ucsd.edu](mailto:beliceiri@health.ucsd.edu), Tel.: +1-858-534-9443,
ORCID: <https://orcid.org/0000-0003-1811-1916>

**Additional Methods**

**qRT-PCR of EV biogenesis gene**

Total RNA was extracted using Trizol reagent (#15596018, Ambion) to evaluate the expression of EV biogenesis genes. The concentration of RNA was quantified by Nanodrop 2000 (Thermo Fisher Scientific), and 1 μg was reversed transcribed using the iScript cDNA synthesis kit (Bio-Rad) on Mycycler (Bio-Rad). The cDNA was used to quantify EV gene expression by real-time quantitative PCR (CFX96, Bio-Rad) in a 25 ml reaction containing 100nM of primers and iQ SYBR Green Supermix (#172-5271, Bio-Rad). The following genes of interest using the housekeeping gene glyceraldehyde-3-phosphate dehydrogenase (GAPDH) as an internal control. Rab5a (QT00084385), Rab5b (QT02248967), Rab27a (QT00040054), Rab27b (QT00006965), Rab11 (QT00241906), Vps4a (QT00022029), TSG101 (QT00090363), and Rab35 (QT00089551) were purchased from Qiagen. The amplification Ct for the genes was normalized to that of GAPDH in each sample, and samples were assessed in triplicate. Results were expressed as mean of at least 3 independent experiments

**Measurement of lentivirus concentration by GoStix**

The Lenti-vpak packaging kit (#TR30037, OriGene Technologies Inc, Rockville, MD, USA) was used for virus production, with lentivirus being collected and concentrated from conditioned media using the Lenti concentrator (#TR30026, OriGene Technologies Inc) and quantified using Lenti-X GoStix Plus (#631280, TaKaRa Bio USA Inc, San Jose, CA, USA) that measures the expression of lentiviral p24 protein using GoStix Value software (Takara). The quantitative output from the Lenti-X GoStix App (downloaded “GoStix Plus”app) was used to compare virus amounts between different preparations.

**Immunofluorescence**

Whole skin tissue samples were collected and fixed with 4 % paraformaldehyde in PBS for 24 hours before embedding in Tissue-Tek Optimal Cutting Temperature (O.C.T. medium (#4583, Sakura) and preparation of 10 μm thick sections. Tissue sections were permeabilized with 0.3 % PBS-TX (Triton X-100) buffer and blocked with 5% BSA for 1 hour at room temperature. Slides were incubated with anti-cytokeratin 14 antibody (#10143-1-AP, Proteintech, IL, USA) diluted 1:400 in PBS/1%BSA overnight at room temperature, washed three times with PBS and incubated with secondary antibody (Goat anti-Rabbit IgG Alexa Fluor™ 488, #A-11008, Thermo Fisher, USA) diluted 1:1000 for 1 hour at room temperature. After washing with PBS, slides were counterstained with DAPI, mounted, and examined by confocal microscopy.

**Additional Figures**


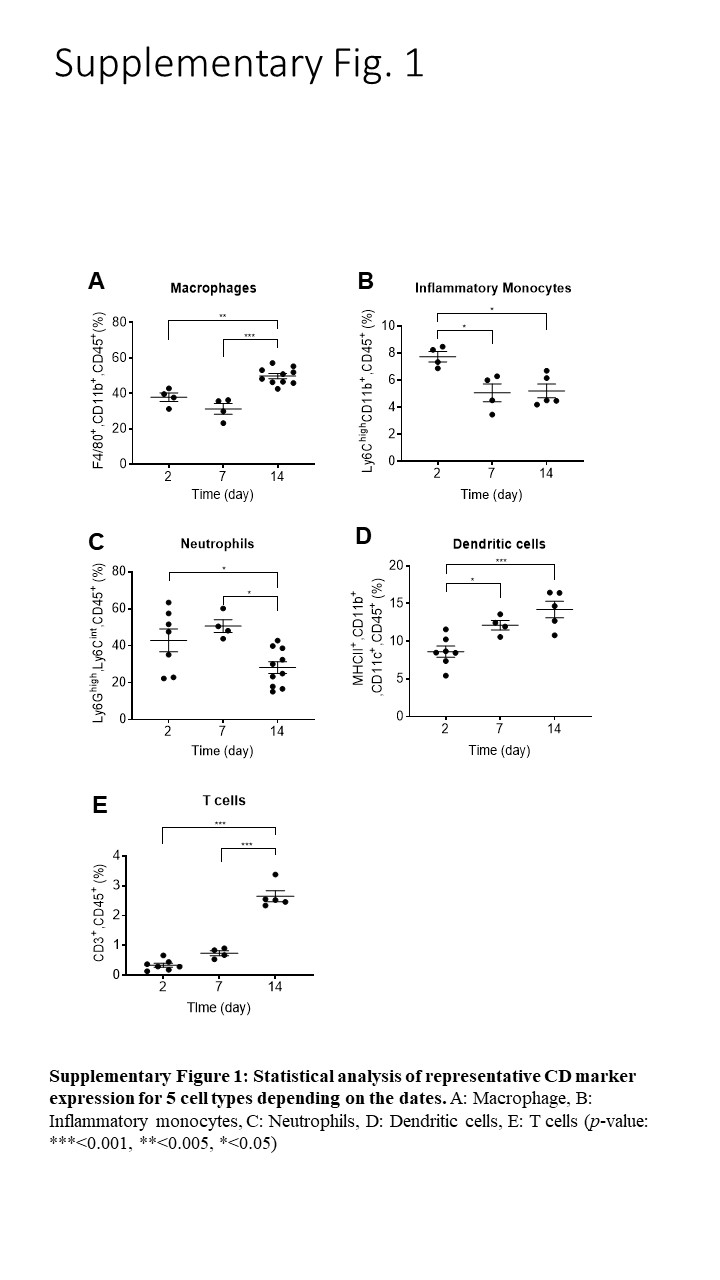


**Figure S1.** **Statistical analysis of CD marker expression in PVA infiltrates at various time points.** In support of the piechart analyses in Fig. 1, immune cells infiltrating PVA sponges were quantified at each time point focusing on: **a** Macrophages, **b** Inflammatory monocytes, **c** Neutrophils, **d** Dendritic cells, and **e** T cells (*p*-value: ***<0.001, **<0.005, *<0.05)


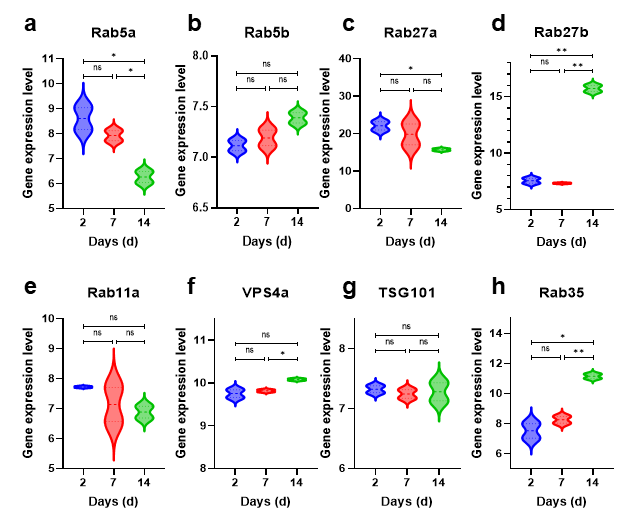


**Figure S2.** **Evaluation of EV biogenesis related gene expression in the PVA cells by qRT-PCR depending on the 2, 7, and 14 days.** Measurements of changes in gene expression normalized to GAPDH in cells infiltrating PVA sponges focusing on the following canonical biogeneis genes: **a** Rab5a, **b** Rab5b, **c** Rab27, **d** Rab27b **e** Rab11a, **f** VPS4a, **g** TSG101, and **h** Rab35 (p-value: **<0.005, *<0.05).


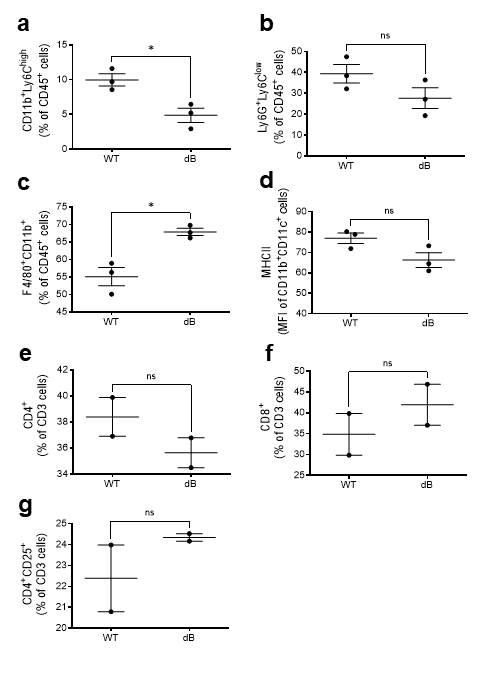


**Figure S3. Analysis of baseline cell infiltrate in PVA sponges of WT vs. db/db mice, from which EVs were harvested.** Flow cytometry analysis of immune cells recruited to the PVA sponge at 14 days post-implantation focusing on : **a** monoyctes based on CD11b^+^Ly6C^high^ cells as a subset of CD45^+^ cells, **b** neutrophils based on Ly6G^+^Ly6C^low^ cells as a subset of CD45^+^ cells, **c** macrophages based on CD11b^+^F4/80^+^ cells as a subset of CD45^+^ cells, **d** DCs based on MHCII^+^ cells as a subest of CD11b^+^CD11c^+^ cells, **e** T cells based on CD4^+^ cells as a subset of CD3^+^ cells, **f** CD8^+^ T cells as a subset of CD3^+^ cells, and **g** Regulatory T cells based on CD4^+^CD25^+^ as a subset of CD3^+^ cells. (*p*-value: *<0.05).


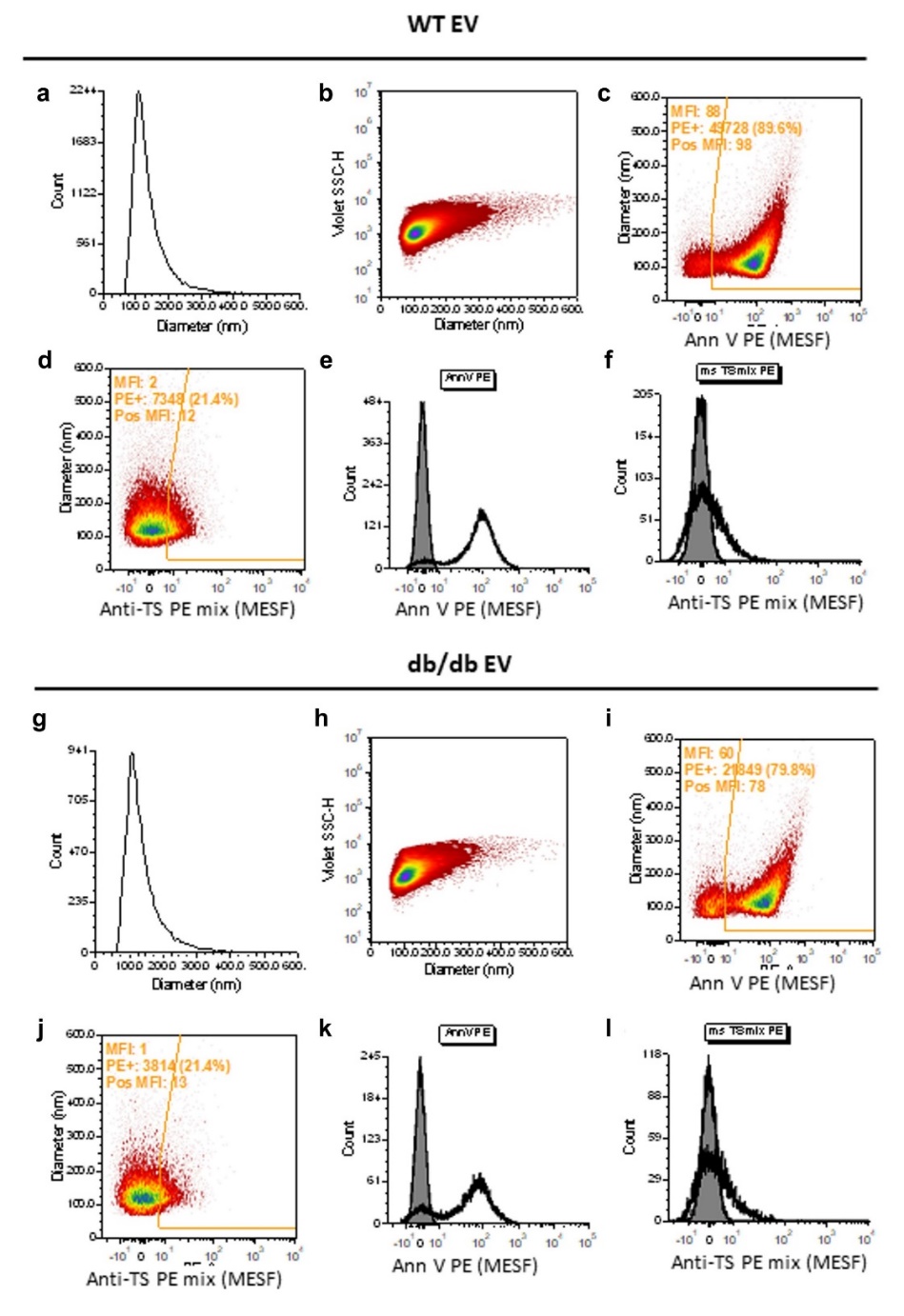


**Figure S4. Validation of EVs derived from WT and db/db mice by vFRed analysis.** **a** Size distribution of WT EVs. **b** Plot diameter data of WT EVs. **c** Expression of Annexin V-PE on WT EVs. **d** Expression of tetraspanins CD9, CD63 and CD81 (TS-PE)on WT EVs. **e** Histogram of Annexin V-PE expression on WT EVs normalized to buffer only (grey filled in). **f** Histogram of TS-PE on WT EVs normalized to buffer only (grey filled in). **g** Size distribution of db/db EVs. **h** Plot diameter data of db/db EVs. **i** Expression of Annexin V-PE on db/db EVs. **j** Expression of TS-PE on db/db EVs. **k** Histogram of Annexin V-PE expression on db/db EVs normalized to buffer only (grey filled in). **l** Histogram of TS-PE on db/db EVs normalized to buffer only (grey filled in).


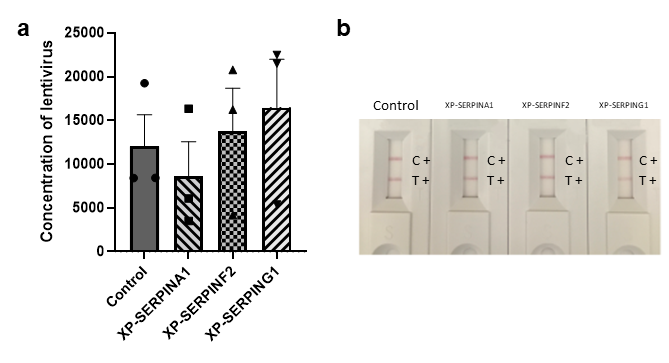


**Supplementary Figure S5. Validation of lentivirus titer testing using HEK293 cells.** **a** Representative comparison of lentivirus yields **b** based on titer testing of lentiviral p24 protein using GoStix analysis per manufacturers recommendations.

**
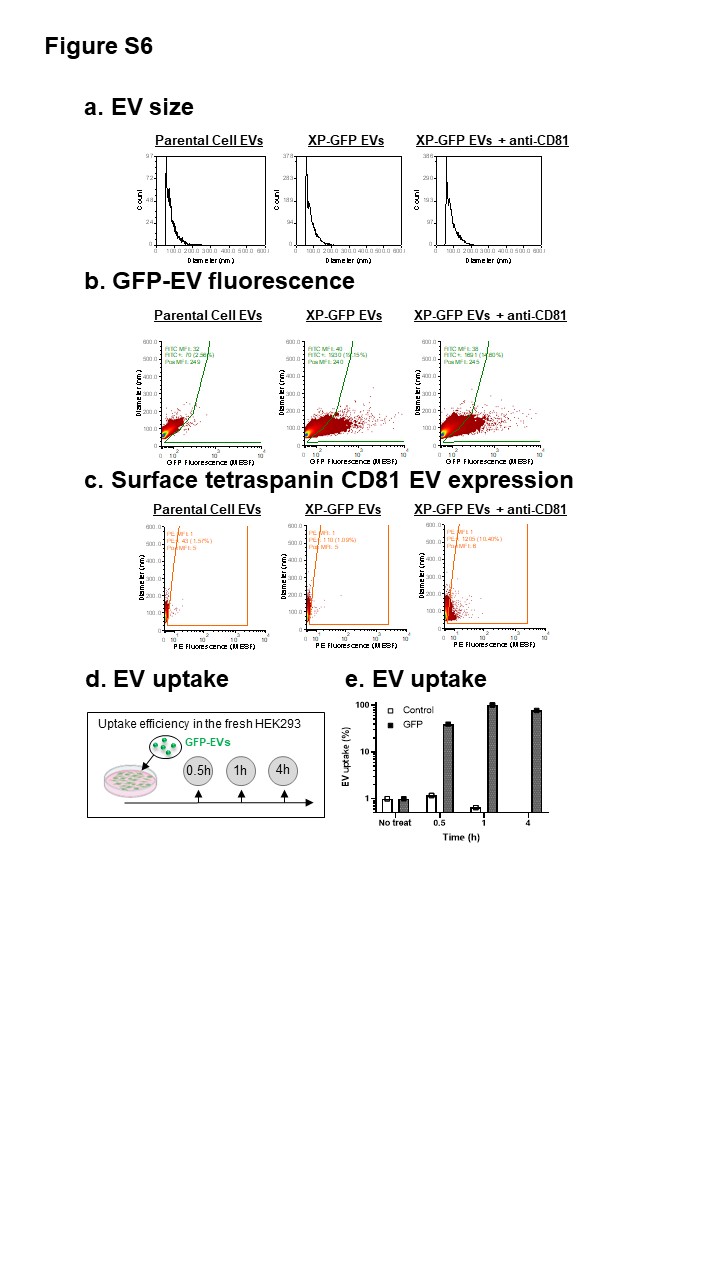
**

**Supplementary Figure S6. A representative example of the analysis of EVs engineered to express specific proteins.** **a** EVs collected from the conditioned media of parental cells as a negative control (Left, naïve HEK293 donor cells) and enriched by density ultracentrifugation were subjected to vFRED staining to determine EV diameter as described in the Materials and Methods. GFP-loaded EVs (XP-GFP) in absence (Left) or presence (Right) of anti-CD81-PE tetraspanin were analyzed for **b** GFP fluorescence and **c** expression of CD81. **d and e** An overview and quantification of GFP-EV internalization into HEK293 cells (filled in) compared to buffer control (open).

**Supplementary Figure S7. Quantification of K14 immunohistochemistry.** The fluorescent intensity of ant-K14 stained tissue sections from Figure 5l were quantified using Image J. A minimum of 3 fields were analyzed for each of the EV treated samples (*p*-value: * <0.05).


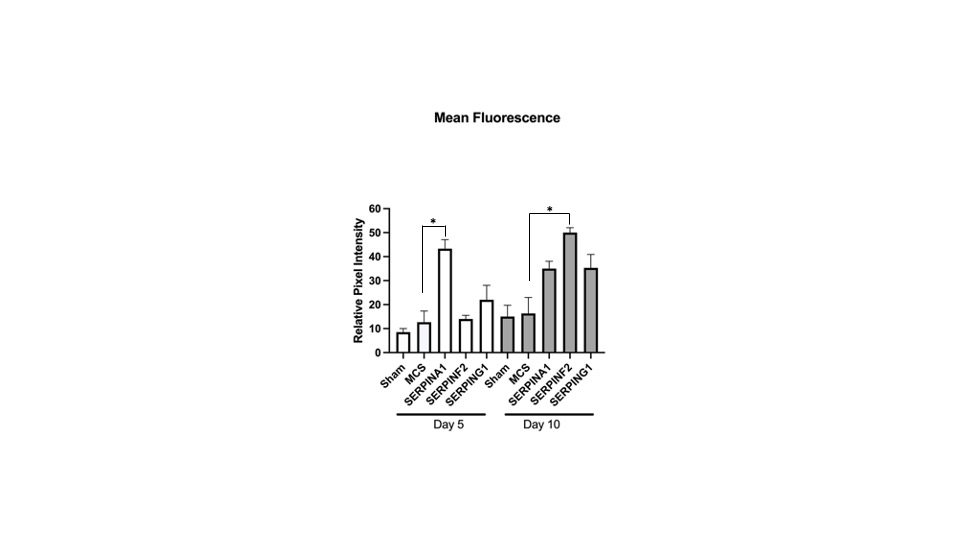


**Additional Tables**

**Table S1.** Detail information of primer for extracellular vesicle biogenesis related genes.

| **Primer name** | **Catalog number** | **Vender** |
| --- | --- | --- |
| Rab5a | QT00084385 | Qiagen |
| Rab5b | QT02248967 | Qiagen |
| Rab27a | QT00040054 | Qiagen |
| Rab27b | QT00006965 | Qiagen |
| Rab11a | QT00241906 | Qiagen |
| VPS4a | QT00022029 | Qiagen |
| TSG101 | QT00090363 | Qiagen |
| Rab35 | QT00089551 | Qiagen |

**Table S2.** Primer sequences for cloning of XP tag in-frame with SERPINA1, SERPINF2, and SERPING1.

| **Primer name** | **Primer sequence** |
| --- | --- |
| F-SERPINA1 | 5’- GCA AAG ATG CCT CGA GGA TGC CGT CTT CTG TCT CGT G -3’ |
| R-SERPINA1 | 5’- AGA ATT CTC GCG GCC GCT TAT TTT TGG GTG GGA TTC ACC AC -3’ |
| F-SERPINF2 | 5’- GCA AAG ATG CCT CGA GGA TGG CGC TGC TCT GGG G -3’ |
| R-SERPINF2 | 5’- AGA ATT CTC GCG GCC GCT CAC TTG GGG CTG CCA AAC TGG -3’ |
| F-SERPING1 | 5’- GCA AAG ATG CCT CGA GGA TGG CCT CCA GGC TGA CC -3’ |
| R-SERPING1 | 5’- AGA ATT CTC GCG GCC GCT CAG GCC CTG GGG TCA TAT ACT CG -3’ |
